# Adaptive Evolution of Phosphorus Metabolism in *Prochlorococcus*

**DOI:** 10.1128/mSystems.00065-16

**Published:** 2016-11-15

**Authors:** John R. Casey, Adil Mardinoglu, Jens Nielsen, David M. Karl

**Affiliations:** a Daniel K. Inouye Center for Microbial Oceanography, Research and Education, School of Ocean and Earth Science and Technology, University of Hawaii, Honolulu, Hawaii, USA; b Department of Biology and Biological Engineering, Chalmers University of Technology, Gothenburg, Sweden; c Science for Life Laboratory, KTH—Royal Institute of Technology, Stockholm, Sweden; d Novo Nordisk Foundation Center for Biosustainability, Technical University of Denmark, Lyngby, Denmark; City of Knowledge

**Keywords:** *Prochlorococcus*, evolution of metabolic networks, flux balance analysis, metabolic modeling, phosphorus metabolism, succinate dehydrogenase

## Abstract

Inorganic phosphorus is scarce in the eastern Mediterranean Sea, where the high-light-adapted ecotype HLI of the marine picocyanobacterium *Prochlorococcus marinus* thrives. Physiological and regulatory control of phosphorus acquisition and partitioning has been observed in HLI both in culture and in the field; however, the optimization of phosphorus metabolism and associated gains for its phosphorus-limited-growth (PLG) phenotype have not been studied. Here, we reconstructed a genome-scale metabolic network of the HLI axenic strain MED4 (*i*JC568), consisting of 568 metabolic genes in relation to 794 reactions involving 680 metabolites distributed in 6 subcellular locations. *i*JC568 was used to quantify metabolic fluxes under PLG conditions, and we observed a close correspondence between experimental and computed fluxes. We found that MED4 has minimized its dependence on intracellular phosphate, not only through drastic depletion of phosphorus-containing biomass components but also through network-wide reductions in phosphate-reaction participation and the loss of a key enzyme, succinate dehydrogenase. These alterations occur despite the stringency of having relatively few pathway redundancies and an extremely high proportion of essential metabolic genes (47%; defined as the percentage of lethal *in silico* gene knockouts). These strategies are examples of nutrient-controlled adaptive evolution and confer a dramatic growth rate advantage to MED4 in phosphorus-limited regions.

**IMPORTANCE** Microbes are known to employ three basic strategies to compete for limiting elemental resources: (i) cell quotas may be adjusted by alterations to cell physiology or by substitution of a more plentiful resource, (ii) stressed cells may synthesize high-affinity transporters, and (iii) cells may access more costly sources from internal stores, by degradation, or by petitioning other microbes. In the case of phosphorus, a limiting resource in vast oceanic regions, the cosmopolitan cyanobacterium *Prochlorococcus marinus* thrives by adopting all three strategies and a fourth, previously unknown strategy. By generating a detailed model of its metabolism, we found that strain MED4 has evolved a way to reduce its dependence on phosphate by minimizing the number of enzymes involved in phosphate transformations, despite the stringency of nearly half of its metabolic genes being essential for survival. Relieving phosphorus limitation, both physiologically and throughout intermediate metabolism, substantially improves phosphorus-specific growth rates.

## Abstract (precis)

Microbes are known to employ three basic strategies to compete for limiting elemental resources: (i) cell quotas may be adjusted by alterations to cell physiology or by substitution of a more plentiful resource, (ii) stressed cells may synthesize high-affinity transporters, and (iii) cells may access more costly sources from internal stores, by degradation, or by petitioning other microbes. In the case of phosphorus, a limiting resource in vast oceanic regions, the cosmopolitan cyanobacterium *Prochlorococcus marinus* thrives by adopting all three strategies and a fourth, previously unknown strategy. By generating a detailed model of its metabolism, we found that strain MED4 has evolved a way to reduce its dependence on phosphate by minimizing the number of enzymes involved in phosphate transformations, despite the stringency of nearly half of its metabolic genes being essential for survival. Relieving phosphorus limitation, both physiologically and throughout intermediate metabolism, substantially improves phosphorus-specific growth rates.

## INTRODUCTION

The picocyanobacterium *Prochlorococcus marinus* is the numerically dominant photoautotroph in the vast oligotrophic gyres, where it often contributes a majority of carbon fixation (1). Ecotypes of the *Prochlorococcus* lineage occupy a broad ecological niche space, and its success has been attributed to its small size, a highly streamlined and nearly minimal genome, and physiological adaptations to low-nutrient environments (2). Natural populations and laboratory isolates adjust their elemental quotas widely in response to nutrient supply by a variety of intriguing mechanisms. Among other notable adaptations, they are capable of utilizing organic substrates to supplement inorganic nutrient deficits (3–5), replacing phospholipids with sulfolipids and glycolipids under phosphate-depleted conditions (6), and coordinating proteome-wide control of nitrogen allocation under nitrogen stress (7). The first axenic strain of the *e*MED4 high-light-ecotype lineage cultivated, MED4 (and synonymous genotypes PCC9511, CCMP1378, and CCMP1986 [8]), has been the subject of numerous physiological studies and was originally isolated from the chronically phosphate-depleted surface waters of the eastern Mediterranean Sea, where it is numerically dominant (9, 10). The phosphorus stress response has been described in detail for MED4 regulation of gene expression (11, 12) and for physiological responses of the cell cycle (13), elemental composition (14), phosphorus substrate uptake rates (15), alkaline phosphatase activity (3), and lipid composition (6). The regulatory circuit includes upregulation of the sensor kinase complex *phoBR* and subsequent upregulation of the high-affinity phosphate transporter system *pstABCS* and the *pho* operon, including alkaline phosphatase. We hypothesized that, in addition to these regulatory and physiological responses, MED4 has optimized, through adaptive gene loss (16), its metabolic network to cope with low phosphate availability. To examine this, we sought a quantitative method to predict the metabolic capabilities of the MED4 genotype and its phosphorus-limited-growth (PLG) phenotype.

Genome-scale metabolic (GEM) network reconstructions represent a cornerstone of systems biology, serving as both a knowledge base for contextualizing physiological and multiomics data types and as a framework for computational approaches, such as constraint-based modeling (17, 18). GEMs are available for a broad spectrum of microbes and model organisms, ranging widely in network size, complexity, and quality (based on the scoring criteria of Thiele and Palsson [19]). Quantitative metabolic flux predictions using constraint-based flux balance analysis (FBA) have been validated experimentally for a range of different organisms. Furthermore, FBA and related approaches have proven valuable for strain engineering, natural product yield optimization, identification of inhibition targets for drug therapies, and numerous other industrial and medical applications (20). Despite these routine applications, to our knowledge, no ecological applications have been reported. Indeed, GEMs of ecologically relevant microbes could complement trait-based and cellular-resource-allocation models, which benefit from broader taxonomic coverage, and could perhaps be nested in global biogeochemical models.

Here, we discuss how we reconstructed a GEM for MED4 (*i*JC568) and used the model to simulate growth in a variety of defined media to describe its metabolic capabilities and quantify fluxes associated with conditions of phosphate-, carbon-, and light-limited growth (PLG, CLG, and LLG, respectively). The imprint of adaptive evolution under phosphate-depleted conditions was found throughout the MED4 metabolic network, with implications for global cellular elemental turnover and energy metabolism. To explore MED4 metabolism, we first described fundamental properties of the metabolic network in relation to a diverse selection of microbial GEMs (referred to below as the ensemble). We then used FBA to compute its metabolic capabilities and compared model simulations with experimental data from both the laboratory and the field. Finally, we discuss genome-wide alterations to phosphorus metabolism and implications for the PLG phenotype.

## RESULTS

### Reconstruction of *i*JC568 and its computing performance.

We reconstructed the GEM for *Prochlorococcus marinus* strain MED4, termed *i*JC568, which consists of 568 metabolic genes encoding 794 reactions with 680 metabolites distributed among 6 subcellular locations (cytoplasmic membrane, periplasm, thylakoid membrane, thylakoid lumen, cytoplasm, and carboxysome) (see Data Set S1 in the supplemental material). A summary of *i*JC568 network properties is given in Table 1, and a comparison with the ensemble is given in Data Set S2.

10.1128/mSystems.00065-16.5 Data Set S1  *i*JC568 model in Excel format. BioOpt format and RAVEN SBML format are available for download at http://biomet-toolbox.org/. Download Data Set S1, XLS file, 0.5 MB. Copyright © 2016 Casey et al. 2016 Casey et al. This content is distributed under the terms of the Creative Commons Attribution 4.0 International license.

10.1128/mSystems.00065-16.6 Data Set S2  Accompanying data sets used and produced in the manuscript. *readme*, description of each data set, as well as hyperlinks to navigate; *BOF*, molar and mass-based composition of crude biomass fractions and their individual components; *Elemental stoichiometry*, comparison of BOF elemental stoichiometry with experimental data; *Enthalpy of combustion*, heats of combustion for each of the biomass components on a molar and carbon molar basis; *CLG Sensitivity*, carbon-limited growth biomass sensitivity; *LLG Sensitivity*, light-limited growth biomass sensitivity; *PLG Sensitivity*, phosphorus-limited growth biomass sensitivity; *NLG Sensitivity*, nitrogen-limited growth biomass sensitivity; *repMets vs Shadow*, table of the top 10 most positive and negative shadow prices for PLG conditions and their corresponding *Z* scores from the reporterMetabolites algorithm; *Rate validations*, comparison of growth rates, photosynthetic parameters, and internal fluxes for *i*JC568 and experimental data; *Gene information*, annotations and identifiers for each metabolic gene included in *i*JC568, gene length, strand sense, whether the gene belongs to the core or flexible *Prochlorococcus* pangenome, the expression level from Wang et al. (29), and the gene product molecular weight and isoelectric point; *Gene essentiality*, results from *in silico* gene knockouts (this worksheet includes essential and nonessential genes from the autotrophic and mixotrophic growth simulations; mixotrophic growth includes a third classification for “variable” essential genes which were lethal deletions only in certain medium compositions); *Ensemble Models*, table summarizing ensemble models (number of metabolites, reactions, metabolic genes, total genes, and essential genes); *Intracellular P_i_*, table of intracellular phosphate concentrations in P-replete and P-limited media for *Prochlorococcus marinus* MED4, *Synechococcus* WH7803, *Escherichia coli* MG1655, and *Saccharomyces cerevisiae*; *Succinate costs in mutants*, table of NAD(P)H costs associated with *de novo* succinate synthesis for each of the strain variants (WT, +SDH, +2OGDC+SSADH, and +2OGDC+SSADH+SDH). Values for NAD(P)H-consuming reactions represent the difference between fluxes in the steady-state solution and fluxes in the forced-accumulation solution. Download Data Set S2, XLS file, 0.4 MB. Copyright © 2016 Casey et al. 2016 Casey et al. This content is distributed under the terms of the Creative Commons Attribution 4.0 International license.

**TABLE 1:** Summary of *i*JC568 properties

Feature^a^	No. (% of total)
Genes	568
Complexed	302 (53)
Reactions	794
Blocked	23 (3)
Orphaned	3 (<1)
Gap filled	60 (8)
Reversible	329 (42)
Transport	63 (8)
Exchange	79 (10)
Metabolites	680
Unique	597 (88)

a Complexed, subunit-encoding genes; Blocked, reactions associated with dead-end metabolites; Orphaned, reactions not connected to the network; Gap filled, metabolic reactions with no annotated gene; Transport, including diffusive reactions and porins; Exchange, boundary transport used for modeling.

To verify the *i*JC568 biomass objective function (BOF; see Materials and Methods) composition, mass and energy budgets, elemental stoichiometry, and standard enthalpies were calculated and compared with reported experimental data. The elemental stoichiometry of the BOF composition was within the standard error of reported values for carbon, nitrogen, and phosphorus ratios under balanced-growth conditions (see Data Set S2 in the supplemental material) (14). We calculated the heats of combustion (21) for each of the 121 compounds comprising the BOF (see Data Set S2). By comparing these values to their energy cofactor demands (calculated as the sum of nucleotide triphosphate, nicotinamide dinucleotide, and flavin adenine nucleotide standard enthalpies), a slope of 29.5 kJ [mol ATP]^−1^ was found, which is quite similar to the theoretical standard enthalpy of ATP hydrolysis (30 kJ [mol ATP]^−1^) (22). The resulting aggregate energy density of MED4 was 28 kJ g ash-free dry weight (DW)^−1^, comparable with the aggregate energy densities of *Escherichia coli* (23 kJ g DW^−1^) and *Saccharomyces cerevisiae* (21 kJ g DW^−1^) (23).

**FIG 1:**
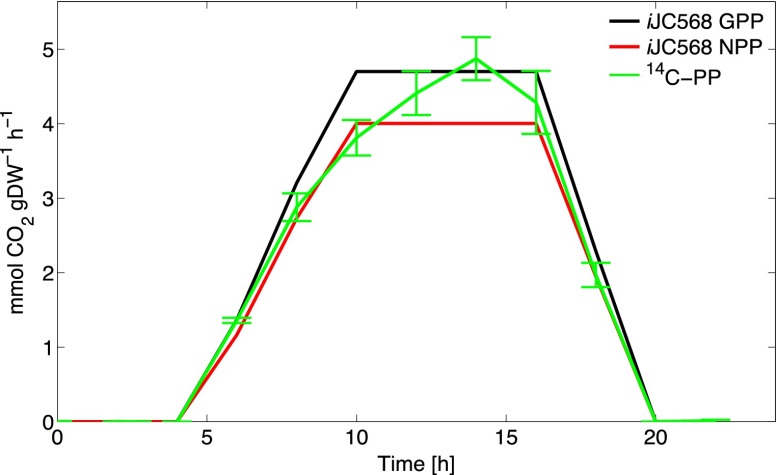
Diel simulation. Comparison of calculated net and gross primary production against short-term [^14^C]bicarbonate primary production measurements reported in reference 26. The light profile followed a gradual increase from darkness to a peak irradiance of 232 µmol photons m^−2^ s^−1^, which was held constant for 4 h, followed by a gradual decrease to darkness.

We verified the FBA results by comparing simulated growth rates, exchange fluxes, and internal fluxes with experimental data by simulating experimental conditions. Growth rates were compared with the results of a fairly extensive set of culture experiments grown on a broad selection of defined-medium compositions and light profiles. The most commonly reported growth condition was a 14-h/10-h light/dark cycle at 20 to 24°C, reaching a peak irradiance ranging from 10 to 56 µmol photons m^−2^ s^−1^ blue light (24–27) in PRO99 medium. Zinser et al. (26) provided the most comprehensive data set relating carbon fixation rates and photophysiology parameters to growth rates, with sampling intervals (2 h) most relevant to our instantaneous flux distributions. By simulating their growth conditions over a diel cycle, we calculated an optimal growth rate of 0.62 day^−1^, while the experimental growth rates were 0.62 ± 0.04 day^−1^ (mean ± standard deviation). The short-term [^14^C]bicarbonate primary production measurements fell between model net and gross primary production for most of the light cycle (Fig. 1). Further comparisons of the *i*JC568 photosynthetic parameters (ATP/NADPH yields, quantum yields, photosynthetic quotient, optimal growth irradiance, and net and gross primary production), growth yields, exchange fluxes (protons, CO_2_, bicarbonate, and nutrients), and central carbon metabolism metrics (phosphoglycerate kinase/phosphoglycerate mutase flux and anapleurotic CO_2_ fixation) were in close agreement with those reported for strain MED4, where available, and for *Synechocystis* sp. strain PCC6803 (see Data Set S2 in the supplemental material). However, the tricarboxylic acid (TCA) cycle and photosynthetic electron flow pathways differed considerably from those of *Synechocystis* sp. strain PCC6803 and are discussed below.

### Metabolic genes and their essentiality.

The proportion of MED4 genes encoding metabolic reactions relative to the total number of genes (30%) is significantly higher than the proportions in the GEM ensemble (19% ± 6%, *P* = 1e^−7^), consistent with the loss of many regulatory functions (28). Nearly all (99.3%) of the metabolic genes were expressed under the conditions tested by Wang et al. (29). The 4 unexpressed metabolic genes included, surprisingly, the gene for malate dehydrogenase (PMM1023), two lipid biosynthetic genes, encoding diacylglyceride kinase (PMM0183) and diacylglycerol phosphatidyltransferase (PMM0798), and unsurprisingly, the gene for arsenate reductase (PMM0512). However, it should be noted that these 4 genes showed low but detectable expression levels in natural samples from the North Pacific Subtropical Gyre (30). We compiled essential metabolic gene sets based on photolithoautotrophic growth on minimal medium (31) and on a supplemental medium (including the 39 carbon substrates, 34 nitrogen substrates, and 95 phosphorus substrates predicted to support growth if suitable transporters were present). Simulated single-gene knockouts were performed for each metabolic gene, and we required that enzyme complexes be complete for the corresponding reaction to proceed. The photolithoautotrophic essential gene set consists of 266 genes, or 47% of the metabolic genes. Although most (88%) of the metabolic genes in *i*JC568 belong to the strain-independent “core” of the *Prochlorococcus* pan-genome (compared with 65% of the whole genome), nonlethal genes were enriched (17%) in strain-dependent “flexible” genes compared with the amount of essential genes (8%). A similar pattern was seen for gene essentiality for mixotrophic growth in supplemental medium (see Data Set S2 in the supplemental material), although a further 196 genes (34% of metabolic genes) produced lethal mutants only under specific conditions (termed “variable-essential”). As in the photolithoautotrophic case, nonessential genes were more frequently part of the flexible pangenome (18%) than variable-essential genes (15%) or essential genes (8%). A genetic system remains elusive for *Prochlorococcus marinus*, so individual knockouts are not yet available to validate these results; however, this is likely a conservative estimate since false negatives are likely when using an *in silico* approach. Where available, the essential gene sets of the ensemble (see Data Set S2) ranged from 12% (*Pseudomonas putida* strain KT2440) to 38% (*Synechocystis* strain PCC6803) of metabolic genes, reinforcing the adaptive gene loss hypothesis for *Prochlorococcus* (16). Examples of bacteria with exceptionally high gene essentiality include the obligate parasites *Mycoplasma genitalium* strain G37 (79% of the whole genome) (32) and *Haemophilus influenzae* strain Rd KW20 (47% of the whole genome) (33).

### Role of phosphate in MED4 metabolism.

**FIG 2:**
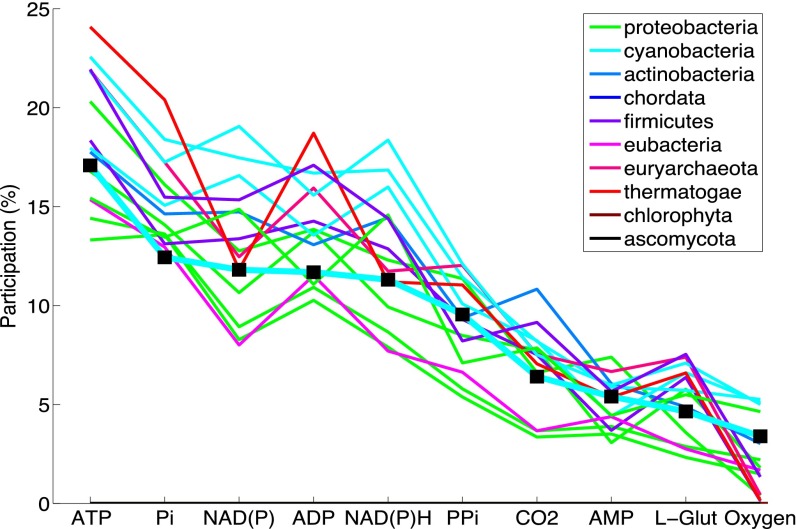
Metabolite participation. Comparison of the 10 highest-degree metabolites (excluding H_2_O and H^+^) between the members of the ensemble, grouped by phylum, and *i*JC568 (■, markers). P_i_, orthophosphate; PP_i_, diphosphate; L-Glut, l-glutamate.

We examined the role of phosphate in MED4 by quantifying its connectivity, dynamic coupling, and turnover within the *i*JC568 metabolic network. A fundamental attribute of the stoichiometric matrix *S* (see Materials and Methods) is the connectivity of the column and row space, defined here as the degree distribution of the undirected bipartite graph. Metabolite participation (i.e., the number of reactions associated with a particular metabolite) was assessed for *i*JC568 and the ensemble by normalizing the degree distribution to the number of nonexchange and transport reactions of each network. For example, the obligate anaerobes *Thermotoga maritima* and *Methanosarcina barkeri* strain Fusaro had oxygen metabolite participation values near zero. The patterns of metabolite participation generally clustered together according to taxonomic group (Fig. 2); however, *i*JC568 deviated from other cyanobacteria for orthophosphate, with the lowest participation among all ensemble models.

The fixed matrix *S* is itself a transformation of the flux vector *v* to the vector of concentration-time derivatives, *dX*/*dt*. Therefore, studying the topology of the matrix *S* and its four fundamental subspaces (the row and null space comprising the flux vector and the column and left null space comprising the vector of concentration-time derivatives) is useful for interrogating network properties and for comparing the structural features and dynamic coupling of networks. Among the numerous factorization methods for analyzing the unconstrained solution space of the stoichiometric matrix, the most intuitive is the singular value decomposition (SVD), given by *S* = *UΣV^T^*. The *m* × *r* column-space (*U*) and *r* × *n* row-space (*V*) matrices contain the linearly independent orthonormal eigenvectors of the four fundamental subspaces of the *m* × *n* matrix *S*, and the singular values *σ* = diag(*Σ*) that define the set {*σ*^1^, … *σ^r^*}, where *r* is the rank of *S*, are measures of the distortion induced by each linear transformation. In this way, the first mode (*σ*^1^) is the weight given to the first systems reaction, a linearly dependent set of reactions forming a basis pathway that is effectively a spanning set of *S*. For *i*JC568, and typical of other networks, the first systems reaction maps to a set of reactions involving proton translocation, due to the central role of the proton motive force in the electron transport chain and photosystems. The first systems reactions correspond quite closely to the metabolites with the highest flux sums (see Materials and Methods), with the exception of the photon flux, which participates in the 3rd and 6th modes in *i*JC568. In other organisms, subsequent modes vary in composition according to the metabolic capabilities of the organism (34). Further analysis of the systems reactions indicated that phosphate metabolism is a less dominant feature of *i*JC568. While the first three modes [ATP hydrolysis, NAD(P) redox, and the proton motive force] were similar for *i*JC568, phosphate transformations were associated with the 8th mode, compared with the 4th mode of *Escherichia coli*, *Haemophilus influenzae*, and *Helicobacter pylori* (34) or the 4th or 5th mode of each of the ensemble models. Accordingly, the fractional singular value (e.g., the percent weight of a particular mode) associated with phosphate transformations was lower in *i*JC568 (0.9%) than in other phyla (range, 1.9% to 6.3%) (see Fig. S1 in the supplemental material).

10.1128/mSystems.00065-16.1 Figure S1  Fractional singular values of the phosphate transformation system reaction for *i*JC568 and the ensemble. References for each ensemble model can be found in Table S2 in the supplemental material. Download Figure S1, EPS file, 0.01 MB. Copyright © 2016 Casey et al. 2016 Casey et al. This content is distributed under the terms of the Creative Commons Attribution 4.0 International license.

The implications of a diminished role for phosphate in MED4 were investigated by comparing the elemental turnover of intermediate metabolism based on flux sums and quotas for hydrogen, carbon, nitrogen, oxygen, phosphorus, and sulfur with that in the high-quality reconstruction (*i*TO977) (35) for *Saccharomyces cerevisiae*. Internal flux sums and turnover were normalized for the differences in optimal growth rate, transport flux of each element, and cellular elemental quotas between iTO977 and *i*JC568. Phosphorus turnover was approximately an order of magnitude higher than that of the other elements in both organisms, but the turnover in *i*TO977 was nearly 3-fold higher than that in *i*JC568 (see Fig. S2 in the supplemental material). Surprisingly little of this turnover (40%) was due to ATP hydrolysis and ADP phosphorylation, with the dissolved P_i_ demand for ATP synthase primarily recycled from the Calvin-Benson-Bassham (CBB) cycle reactions d-glyceraldehyde-3-phosphate:NAD^+^ oxidoreductase and sedoheptulose 1,7-bisphosphate 1-phosphohydrolase. The majority of the remaining 60% of the P turnover was shared between reactions with phosphorylated central carbon metabolites, nucleic acid intermediates, and dinucleotide energy carriers, implying that much of the difference in turnover is due to phosphate participation.

10.1128/mSystems.00065-16.2 Figure S2  Elemental flux sums and turnover comparison of *i*JC568 and *i*TO977. Download Figure S2, EPS file, 0.01 MB. Copyright © 2016 Casey et al. 2016 Casey et al. This content is distributed under the terms of the Creative Commons Attribution 4.0 International license.

### Physiological response to low phosphate.

**FIG 3:**
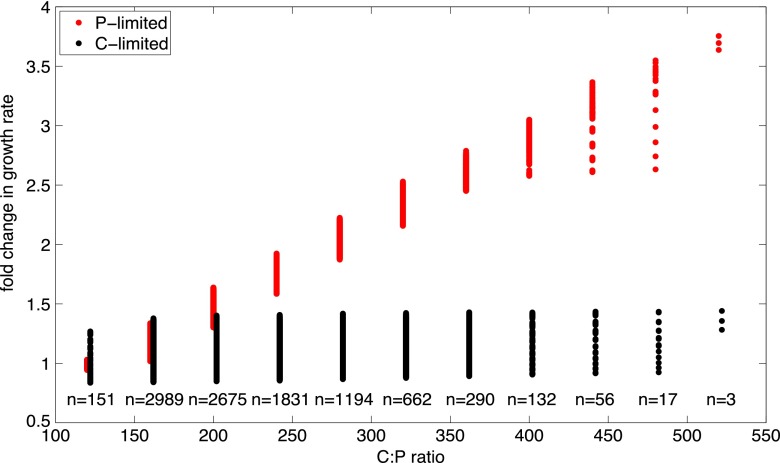
Simulated growth rates as a function of altered biomass compositions. Values represent the calculated growth rates associated with a composition of DNA, RNA, lipid, cell wall, and soluble pool which corresponds to each interval of the C/P ratio range. Growth rates were compared by constraining the orthophosphate transporter flux (red) or the carbon fixation flux (black) to suboptimal rates. The number of biomass compositions at each C/P ratio is indicated (n).

*Prochlorococcus* is known to have an extremely flexible elemental stoichiometry, perhaps a key to coping with the variable supply of nutrients and extended periods of nutrient starvation typically encountered in the oligotrophic surface waters. Populations of *Prochlorococcus* in the periodically P-limited Sargasso Sea exhibited a wide range of particulate C/P ratios (120:1 to 350:1), varying latitudinally (36). When grown in batch culture under P limitation (molar NH_4_^+^/H_2_PO_4_^−^ ratio = 800:1), the MED4 particulate C/P ratio increased to 464:1 ± 28:1, compared with 121:1 ± 17:1 under balanced growth (molar NH_4_^+^/H_2_PO_4_^−^ ratio = 16:1) (14). The partitioning of P in crude fractions of MED4 biomass, calculated by the elemental composition of the BOF, is predominantly bound in RNA (45%), DNA (23%), cell wall (di-*trans*-poly-*cis*-undecaprenyl diphosphate and lipid A disaccharide; 15%), and the soluble pool (BioPool) (especially inorganic P, nucleotides, folate cofactors, and several vitamins; 14%). The remaining P quota (2%) is found in lipids and in protein fractions. Since the discovery that P-limited MED4 and other *Prochlorococcus* strains have virtually eliminated phospholipids (2% of total lipid) in favor of sulfolipids and glycolipids (66% and 32% of total lipid, respectively) (6), the majority remains in the cell wall and nucleotide fractions. DNA-P is static throughout the G_1_ cell cycle phase, and the whole proteome’s phosphorylation state is unlikely to vary significantly, so it follows that the ability to modulate C/P ratios to such extremes (~464:1) requires that all of these fractions must be capable of drastic reductions. Accounting for the 33% increase of the C quota under P limitation (14), the cumulative P quota in non-DNA pools (lipid, protein, RNA, cell wall, and the soluble pool) must be reduced by 85% to achieve a C/P of 464:1, and the additional constraint of genome replication exacerbates this problem. MED4 must therefore regulate C/P ratios beyond those reached by lipid head-group substitution alone; such a reduction undoubtedly has profound impacts on cellular metabolism and physiology. An exhaustive search (see Materials and Methods) was implemented to quantify the growth rate advantage imparted to the PLG and CLG phenotypes by varying crude fractions of biomass to meet a range of feasible cellular C/P ratios (Fig. 3). Over the allowable range of C/P ratios (120:1 to 528:1), the changes in growth rates for the CLG phenotype (14% ± 7%) were identical (*P* = 0.71) to the coefficients of variation (CV) within any particular biomass composition (12% to 14%). In contrast, the growth rates increased 370% ± 12% over the allowable C/P range for the PLG phenotype, with smaller compositional variations (CV = 2% to 9%; two-sample *F* test, *P* ≤ 1e^−6^). To identify which biomass components would yield the highest growth rate gains, we performed a brute-force sensitivity analysis (Ψ*_k_*) (see Materials and Methods; see also Data Set S2 in the supplemental material). Positive Ψ*_k_* values imply an increase in growth rate resulting from a unit decrease in a particular biomass precursor pool *k* or an individual compound within a specified biomass precursor pool *k*. Among the crude biomass fractions, DNA, RNA, lipid, and cell wall were responsible for 96% of the growth rate sensitivity. Since DNA content is considered static in G_1_ phase, the crude fractions with the highest growth sensitivities were RNA (Ψ*_k_* = 0.45), cell wall (Ψ*_k_* = 0.15), and the soluble pool (Ψ*_k_* = 0.13). Within the cell wall crude fraction, di-*trans*-poly-*cis*-undecaprenyl diphosphate and lipid A disaccharide were responsible for 74% and 26% of the sensitivity, respectively. Within the soluble pool, most (70%) of the sensitivity was due to nicotinamide dinucleotides.

Differential gene expression may be used to infer changes in metabolism using the reporter metabolite and reporter subnetwork algorithms (see Materials and Methods). By ranking *Z* scores for each reporter metabolite, we identified a set of metabolites which were associated with up- or downregulated proteins from differential expression (11) and changes in protein abundance (37) of phosphorus-limited MED4 cultures. The top reporter subnetworks included lipopolysaccharide (LPS) synthesis, tRNA synthesis, cell wall synthesis, and a large subnetwork associated with the CBB cycle branches for carbon fixation, lower glycolysis, and the reductive pentose phosphate pathway (PPP) (see Fig. S3 in the supplemental material). Similarly, shadow prices (*λ*) (see Materials and Methods) can be used to infer the degree to which the production of certain metabolites is limiting growth. By constraining a suboptimal upper bound on the P_i_ transport rate and calculating the shadow prices, a set of 28 metabolites were determined to be negative, or growth limiting. These include phosphate esters, nucleotides, cell wall precursors, NADH, and the phosphorylated central carbon metabolites 3-phosphoglycerate and 2-phosphoglycolate. A comparison of reporter metabolites and shadow prices showed agreement between *Z* scores and the relative magnitude |− λ| (see Data Set S2). These qualitative (gene expression) and quantitative (shadow prices) predictions are complementary but independent methods and were partially validated in our laboratory comparison of the amounts of LPS in culture (see Materials and Methods), where P-limited cells showed a 55% ± 4% reduction compared with P-replete cells.

10.1128/mSystems.00065-16.3 Figure S3  Reporter subnetworks identified from differential expression in P-replete and P-limited media. Data are from reference 11. Download Figure S3, JPG file, 1.8 MB. Copyright © 2016 Casey et al. 2016 Casey et al. This content is distributed under the terms of the Creative Commons Attribution 4.0 International license.

**FIG 4:**
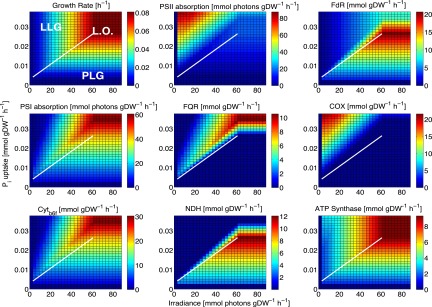
Phenotype phase planes of light and phosphate uptake for key photosynthetic fluxes. In each panel, the white line of optimality (L.O.) indicates optimal growth and delineates LLG (above) and PLG (below) phenotypes. FdR, ferredoxin-NADP^+^ reductase; FQR, ferredoxin:quinone oxidoreductase; COX, cytochrome oxidase *bd*; Cyt_*b*6*f*_, cytochrome *b*_6_*f*; NDH, NADPH dehydrogenase type 1.

In culture, P stress induced changes not only in acquisition mechanisms and biosynthetic pathways but also in central carbon metabolic pathways and the photosynthetic apparatus. Following the initiation of P stress, photosystem II (PSII) was degraded, with a concomitant decrease in carbon fixation proteins, although photosystem I (PSI) and subunits of the ATP synthase complex remained intact (37). This finding was also apparent for the *in silico* PLG phenotype in *i*JC568, with an increase in the PSI/PSII photon absorption ratio at the maximum growth irradiance (*I*_max_) as a nonlinear function of the P_i_ transporter flux, converging on a new steady state for ATP and reductant for the PLG phenotype. We compared growth and key photosynthetic fluxes between the LLG and PLG conditions by phenotype phase plane (PhPP) analysis, varying light and P_i_ uptake rates (Fig. 4; see also Fig. S4 in the supplemental material). In *i*JC568, the linear electron flow (LEF) pathway begins with PSII, cytochrome *b*_6_*f* (Cyt*_b_*6*_f_*), and PSI and ends with ferredoxin-NADP^+^ reductase (FdR). LEF is linked by the oxidation and reduction of the plastocyanin (Cu^2+^PC/Cu^+^PC), ferredoxin (Fd_ox_/Fd_red_), and plastoquinone (PQ/PQH_2_) pools. A set of alternative electron flow (AEF) pathways include cyclic electron flow (CEF) around PSI via NADPH dehydrogenase type 1 (NDH) or via ferredoxin:quinone oxidoreductase (FQR), pseudocyclic electron flow (PCEF) around PSII via cytochrome oxidase *bd* (COX; MED4 apparently lacks the *aa*_3_-type cytochrome *c* oxidase), the Mehler reaction, and photorespiration. The activities of AEF pathways affect a number of fundamental fluxes, including the ATP/NADPH ratio, photosynthetic efficiency, quantum yield, and the photosynthetic quotient. Under optimal growth conditions (along the line of optimality [LO]), the ratio of PSI to PSII absorption was 2.3, with the entirety of the PSI flux split between CEF around PSI via NDH to prevent overreduction of the PQ pool and to NADPH via FdR to maintain the optimal ATP/NADPH ratio of 1.30. Under LLG conditions (above the LO), the PSI-to-PSII absorption ratio increased to 2.4, with the PSI flux mostly diverted to NADP^+^, at the expense of CEF, via NDH. Under PLG conditions (below the LO), ATP deficits resulted in a PSI-to-PSII absorption ratio of 0.8, with excess reductant diverted to PCEF around PSII via COX and LEF to NADPH from PSI. CEF around PSI was diverted to FQR from NDH under PLG conditions.

10.1128/mSystems.00065-16.4 Figure S4  Illustration of changes to photosynthetic electron flow under optimal growth conditions, light-limited growth conditions, phosphorus-limited growth conditions, and phosphorus-limited growth conditions for the *in silico* SDH knock-in mutant. The center panel is a detailed view of the *i*JC568 photosystem, including the transport of protons across the thylakoid membrane (orange text), cofactors associated with each reaction (black arrows and numbers), and the stoichiometry of metabolites associated with each reaction (blue arrows and numbers). Reactions belonging to the LEF (pink), CEF (yellow), PCEF (orange), and succinate dehydrogenase knock-in (black) include photosystem II (PSII), ferredoxin-NADP^+^ reductase (FdR), photosystem I (PSI), ferredoxin:quinone oxidoreductase (FQR), cytochrome oxidase *bd* (COX), cytochrome *b*_6_*f*, NADPH dehydrogenase type 1 (NDH), ATP synthase, and succinate dehydrogenase (SDH). Reactions catalyze oxidations (toward the left) and reductions (toward the right) of ferredoxin (Fd_ox_/Fd_red_), plastoquinone (PQ/PQH_2_), NADP^+^/NADPH, and plastocyanin (Cu^2+^/Cu^+^). Arrows in the condition-specific panels (top left, top right, bottom left, bottom right) are scaled by the flux of electrons, based on the individual fluxes, the stoichiometry of each metabolite, and the number of valence electrons exchanged, normalized to the incident number of photons absorbed. Download Figure S4, TIF file, 2.8 MB. Copyright © 2016 Casey et al. 2016 Casey et al. This content is distributed under the terms of the Creative Commons Attribution 4.0 International license.

It is plausible that persistent changes to the optimal path of electron flow under P-limited conditions have resulted in a restructuring of the MED4 photosynthetic apparatus. MED4 lacks the genes encoding succinate dehydrogenase (SDH), which catalyzes the succinate-fumarate couple and directly links the oxidation of TCA cycle intermediates to the reduction of the PQ pool in the photosynthetic electron chain. Succinate oxidation forms an AEF pathway which supplies reductant to Cu^2+^PC at the cost of PQH_2_ via Cyt_*b*6*f*_. Furthermore, all cyanobacteria have a branched TCA cycle, lacking 2-oxoglutarate dehydrogenase; however, *Prochlorococcus marinus* and marine *Synechococcus* spp. also lack the recently discovered analogous enzymes 2-oxoglutarate decarboxylase (2OGDC) and succinic semialdehyde dehydrogenase (SSADH), which are necessary to regenerate succinate from 2-oxoglutarate (38) and have long been implicated in obligate photolithoautotrophy (39). To quantify the effect of SDH and the branched TCA cycle on the energy budget of *i*JC568, we compared the NAD(P)H costs of four *in silico* variants grown photolithoautotrophically: wild type (WT), +2OGDC+SSADH, +SDH, and +2OGDC+SSADH+SDH. NAD(P)H costs were calculated as the change in flux sums for each variant after forcing a net accumulation of succinate (*dX_i_*/*dt* > 0, where *i* corresponds to succinate). For both the WT and the +2OGDC+SSADH mutant, the NAD(P)H cost of succinate synthesis was primarily due to the CEF enzyme NDH and the Calvin-Benson-Bassham pathway enzyme triosephosphate dehydrogenase. For both the +SDH and +2OGDC+SSADH+SDH mutants, electron flow largely bypasses NDH, reducing the cost of succinate synthesis. However, for the +SDH mutant (lacking the cyanobacterial-type TCA cycle), additional NAD(P)H costs were required for precursor synthesis via malate dehydrogenase. In summary (details are in Data Set S2 in the supplemental material), the NAD(P)H cost of regenerating succinate *de novo* for mutants with the cyanobacterial TCA cycle increases from 14 to 16 molecules of NAD(P)H in the absence of SDH, while the reverse is true for MED4 WT, in which the cost decreases from 21 to 14 molecules of NAD(P)H in the absence of SDH. These findings are qualitatively in agreement with the findings for wild-type and –SDH, –2OGDC, and –SSADH mutants of *Synechococcus* sp. PCC7002 (38). As a consequence of the MED4 (WT) TCA pathway, under PLG conditions, the absence of SDH reduces the PSI flux by 30%, resulting in a 16% to 37% increase in quantum yield (mol CO_2_ reduced [mol photons]^−1^) over the range of PLG phenotypes.

## DISCUSSION

We explored the metabolic strategies of the *Prochlorococcus* PLG phenotype in an effort to characterize its adaptation to low-phosphate marine environments. In addition to sulfolipid substitution and utilization of organophosphorus to satisfy P demand, we uncovered three additional strategies that reduce the influence of phosphorus control over optimal growth flux topology: (i) choreographed reductions in enzymes reliant on orthophosphate as a substrate across the entire metabolic network, (ii) reductions in phosphorus-rich biomass constituents, and (iii) alterations to photosynthetic and respiratory electron flow. Phosphorus, the “staff of life” (40), plays myriad roles in the structural, regulatory, and energetic functions of all cellular life. The phosphate residue provides hydrophilicity and increased water solubility of the parent chain, provides a charge to prevent membrane permeation, and provides a nucleophile repellent to resist hydrolysis. Phosphoester bonds modulate posttranslational regulation of protein function, and phosphodiester bonds form the connective tissue of the DNA and RNA backbone. The phosphoanhydride bonds of nucleotide polyphosphates and polyphosphates contain the ultimate source of chemical energy required for all metabolism and, once hydrolyzed, the free monomeric metaphosphate ion is a strong electrophile and phosphorylating agent, capable of phosphorylating even aromatic amine rings, attacking carbonyl groups and ketones to yield their enol phosphates. It is, therefore, unsurprising that hydrolysis, esterification, and isomerization of phosphorylated metabolites are ubiquitous functions in metabolic networks. Since typical intermediate metabolite pool concentrations meet or exceed the associated enzyme half-saturation constant for growth in rich medium (41), a phosphorus-limited metabolic state would, presumably, influence substrate binding kinetics widely. At the level of pathways, phosphate availability would distribute rate limitation broadly, in keeping with metabolic control analysis (42), which finds a nonzero elasticity coefficient for almost all enzymes, resulting in flux control shared among all participating reactions in a pathway.

A strategy to alleviate metabolic control of a single, persistent rate-limiting cosubstrate might be to selectively reduce its degree (“participation”) in the network. In a comparison between *i*JC568 and the ensemble, phosphate participation was lowest for *i*JC568. Reductions in phosphate participation result in a greatly diminished “role” (fractional contribution of the phosphate transformation system reaction to the singular value spectrum) for phosphate from a network perspective, suggesting that low phosphate availability may have guided gene loss during the evolution of MED4, streamlining the loss of genes associated with phosphate transformations. Low-phosphate-reaction participation in *i*JC568 contributed to decreased total elemental phosphorus fluxes in a comparison with *i*TO977, resulting in slower turnover and possibly lowering soluble phosphate concentration requirements to maintain optimal fluxes throughout the network. This prediction is supported by experimental determinations of intracellular phosphate concentrations and their responses to phosphate availability (see Data Set S2 in the supplemental material). Under P-replete conditions, the intracellular phosphate concentrations were sevenfold lower in MED4 than in another oligotrophic marine picocyanobacterium, *Synechococcus* WH7803 (43). Importantly, when grown in P-depleted medium, MED4 intracellular phosphate decreased by only 22% ± 3%, compared with 69% ± 4% for *Synechococcus* WH7803. A similar contrast might be made between MED4 and *Saccharomyces cerevisiae* (44), although the experimental conditions could not be directly compared. To our knowledge, this is the first example of nutrient control of metabolic network evolution.

Beyond the inherent architecture of the metabolic network, MED4 is known to respond physiologically to low phosphate availability by widely altering its elemental C/P ratio. The range of the flexible elemental stoichiometry of MED4 in culture and in the field presents a problem if carbon quotas increase by only 20% and C/P ratios approach the theoretical limit of 528:1 under severe phosphorus limitation (C/P = 464:1): how can genome replication be feasible when a fully replicated genome alone translates to a C/P of 264:1? Based on comparison of the phosphatidylglycerol contents of P-replete and P-limited cultures (45), substitution of sulfo- and glycolipids for the phospholipid head group accounts for 2% of the required P quota reduction, so we sought *in silico* methods to identify where the additional 98% of P quota reductions were to be found. Sensitivity analysis of the BOF composition suggested that reductions in RNA and the cell wall were likely candidates. The selective reductions in phospholipid, cell wall, and RNA synthesis were also observed by the method of reporter subnetworks from differential gene expression under balanced- versus phosphate-limited-growth conditions. Our exhaustive search algorithm predicted a set of feasible biomass compositions for a range of C/P ratios, with optimal growth corresponding to a phosphorus composition for DNA/RNA/lipid/BioPool/cell wall of 1:2.8 ± 0.5:4.7 ± 0.9:1.6 ± 0.4:3.0 ± 0.5. This optimal biomass composition was partially validated in our culture experiments with a shift in the DNA/cell wall ratio from 1.51 under CLG conditions to 2.75 under PLG conditions, assuming LPS remains proportional to cell wall content. It is unclear what physiological effects such a dramatic reduction in cell wall might have; even under conditions of rich medium growth, MED4 has a reduced cell wall thickness (19 nm), compared with 34-nm thickness in a strain isolated from deeper in the euphotic zone where phosphate limitation is less prevalent (46).

Consistent with the theme of low-phosphate-guided gene loss, the conspicuous absence of SDH in MED4 and other ecotype HL-I strains prompted us to investigate the role of this otherwise ubiquitous enzyme under a variety of growth conditions. The reversible succinate-fumarate couple and its catalyst, SDH, are found in all three domains of life, including the last universal ancestor, and were probably conserved throughout organismal evolution (47). Additionally, SDH represents a unique connection between the TCA cycle and respiratory and photosynthetic electron flow, and it is thus under considerable evolutionary pressure that *e*HL-I has shed SDH. The loss of SDH creates an unexpected link between photosynthetic quantum yield and phosphorus-limited growth, and it is at least suggestive that the gene coding for the A subunit of SDH, *sdhA*, is the one annotated gene that differentiates the high-light ecotypes *e*HL-I, which dominates the P-limited Mediterranean Sea, and *e*HL-II, which dominates the predominantly N-limited Atlantic and Pacific Oceans.

### Concluding remarks.

Nearly three decades after its isolation, MED4 has very likely undergone considerable laboratory evolution; however, its metabolic potential remains imprinted with the signature of the chronically phosphorus-depleted surface waters of the eastern Mediterranean Sea. Metabolic network reconstruction and constraint-based modeling revealed previously unknown evolutionary strategies for organisms perpetually coping with low phosphate availability. These strategies include a redesign of the metabolic network to alleviate metabolic control by a single substrate, global control of phosphorus partitioning in biomass components, and optimization of photosynthetic electron flow.

## MATERIALS AND METHODS

### Network reconstruction.

A metabolic network of MED4 was created by following the reconstruction process detailed previously (19, 48). Briefly, an initial draft reconstruction was created by identifying protein homology with the Kegg Orthology (KO) database supplied through the BioMet Toolbox (http://biomet-toolbox.org). Hidden Markov models (HMM) of protein sequences for each KO were retrieved and queried against the MED4 reference genome (NCBI GenBank: BX548174.1). Metabolic genes which were excluded from HMM hits were individually examined using different resources (NCBI, UniProt, IMG, BioCyc, and ProPortal). General and unbalanced reactions were excluded, and extensive manual curation was performed for the gap-filling and balancing process, due in part to the conservative reaction assignment criteria, as well as the incomplete genome annotation (30% of open reading frames [ORFs] were assigned to putative or unknown functions), which is typical of marine cyanobacteria (e.g., 48% of ORFs are assigned to putative or unknown functions for *Synechocystis* sp. PCC6803). Draft model reactions were checked for elemental and charge balance, for known substrate and cofactor specificity, and for directionality. Reaction directionality was determined by thermodynamic favorability (49), followed by manual inspection and elimination of futile cycles, according to the guidelines described in reference 19. Cofactor specificity, especially with regard to preference for NAD(H) and NADP(H), was often unknown; however, the 4 genes (PMM1127, PMM1145, PMM1146, and PMM1147) encoding the alpha and beta subunits of the reversible membrane-bound nicotinamide nucleotide transhydrogenase (EC 1.6.1.1; R00112) effectively eliminated the need to differentiate these important cofactors. Proteins were assigned to one of six subcellular locations: cytoplasmic membrane, periplasm, thylakoid membrane, thylakoid lumen, cytoplasm, or carboxysome. Protein localization was based on amino acid sequences using the PSORTb algorithm for bacteria (50) and the ExPASy tool DAS-TMfilter for transmembrane domain prediction (51). Proteins associated with the thylakoid membranes and carboxysomes are not predicted by PSORTb and were instead inferred from homology to a detailed photophysiological model for *Synechocystis* sp. PCC6803 (52).

Gaps were identified by iteratively examining dead-end metabolites and associated blocked reactions and returning to the literature for evidence of synthesis, degradation, secretion, or uptake of associated metabolites. Conserved domains from the resulting orphan reactions were then queried against the MED4 genome by protein homology using BLASTp. The resulting well-connected network was then queried for futile cycles, and transport and exchange reactions were added. Several exchange reactions were added for protein complexes (e.g., acyl carrier protein and lipoylprotein) which are not explicitly synthesized by the network, though these carry no flux and are included only for modeling purposes. Fake exchange reactions were also added for dead-end metabolites not included in the biomass objective and for which no transporters are annotated (e.g., glycolaldehyde, 7-aminomethyl-7-carbaguanine, and methanol). These reactions can carry flux and are considered analogous to diffusive transport. A tunable ATP sink was introduced, also for modeling purposes, to account for costs associated with photodamage above an experimentally determined irradiance (549 µmol photons m^−2^ s^−1^) (53), though this reaction is constrained to zero unless explicitly stated otherwise herein. Transporter proteins are particularly poorly annotated in the MED4 genome, so physiological evidence alone was required for transporter presence in some cases. Because transport may variously be chemiosmotic (symporter/antiporter ion pumps) or mediated by ATP hydrolysis, it is likely that *i*JC568 is not accurately charge balanced with respect to major ions (e.g., K^+^ and Ca^2+^). In all cases, the presence/absence of the reaction was scored for evidence according to the guidelines in reference 19.

The process of building an *in silico* metabolic reconstruction is, historically, a series of iterative improvements whereby the model grows in size and complexity, often with detail added to specific pathways as experimental data become available. Open code and computational design are essential to this process, and we have made efforts to enable community contributions. The model is fully MIRIAM compliant and is available in standard formats (SBML for RAVEN and BioOpt at http://biomet-toolbox.org; Excel format in Data Set S1 in the supplemental material). Since naming conventions and database link identifiers differ widely, the Excel file contains additional fields to identify reactions (SBO terms, KEGG Orthology, and EC codes), metabolites (molecular formula, molecular weight, charge, IUPAC name, InChI, InChIKey, PubChem compound identifier [CID], and KEGG compound), and genes (KEGG gene, NCBI accession number, and UniProt identifier [ID]), which are intended to aid in formatting conversions for ease of sharing. Simulation results and the BOF are available as tabs in a separate Excel file (see Data Set S2).

### Constraint-based modeling.

FBA and several related approaches were employed in the manuscript. In the dynamic state, FBA seeks to maximize or minimize a metabolic function, such as biomass growth or ATP dissipation, subject to constraints on fluxes as follows:

(1) $$\begin{array}{c} \text{Minimize}\\ Z=-c^{T}v\\ \text{Subject to}\\ S\cdot v-b=\frac{dX}{dt},\\ v_{j}^{\mathrm{LB}}\leq v_{j}\leq v_{j}^{\mathrm{UB}} \end{array}$$

where *Z* is growth rate, *c* is a vector of coefficients of length *n* identifying the objective reaction in the flux vector *v* of length *n*. *S* is the stoichiometric matrix of metabolites and reactions of dimension *m* × *n*, *b* is a vector of exchange fluxes of length *n*, and *X* is a vector of metabolite concentrations of length *m*. LB and UB refer to the upper and lower bounds on the *j*th reaction in the flux vector *v*. In the steady state, the problem is restated by

(2) $$\frac{dX}{dt}=0$$

implying that there is no net accumulation or depletion of any metabolite pools. The optimization package Mosek (Mosek ApS, Denmark) was used to find the primal solution of the linear programming (LP) problem. Elemental flux sums (Φ*_i_*) were calculated using the elemental matrix *E*, constructed for hydrogen, carbon, nitrogen, oxygen, phosphorus, and sulfur from metabolite molecular formulas as follows:

(3) $$\Phi_{i}=\frac{1}{2}\sum_{j}\left|E_{ij}v_{j}\right|$$

### Shadow prices.

Sensitivity analyses were based on so-called shadow prices of the dual solution to the LP problem according to

(4) $$\sum_{i=1}^{m}\lambda_{i}=\frac{dZ}{dX_{i}}$$

where dual variables λ of length *m* are assigned to steady-state constraints, and variables *q_1_* and *q_2_* are assigned to the flux constraints *v*^LB^ and *v*^UB^, respectively, as follows:

(5) $$\begin{array}{c} \text{Minimize}\\ -q_{1}v^{\mathrm{LB}}-q_{2}v^{\mathrm{UB}}\\ \text{Subject to}\\ c^{T}=\lambda^{T}S+q_{1}^{T}+q_{2}^{T},\\ q_{1}\leq 0,q_{2}\geq 0 \end{array}$$

### Reporter metabolites and reporter subnetworks.

A hypothesis-based method to identify key biological features around which transcriptional changes occur was implemented to interpret the phosphorus stress response, using the algorithms for reporter metabolites (54) and reporter subnetworks (55). Both algorithms map the *P* values and fold changes from a differential expression data set (11) to the metabolic network using gene-protein-reaction associations. The reporter metabolite algorithm ranks metabolite nodes based on the normalized transcriptional response of its neighboring protein nodes according to *Z* scores assigned to each edge. The reporter subnetwork algorithm expands on this concept by randomly sampling aggregates of reporter nodes and, again, ranking each aggregate according to its *Z* score.

### Biomass objective function.

A detailed biomass objective function (BOF) is essential to any high-quality GEM. *i*JC568 includes detailed biomass composition data collated from the MED4 literature, under similar growth conditions (PRO99 medium, 14-h/10-h light/dark cycle with peak intensities of 40 to 80 µmol photons m^−2^ s^−1^) where available. Our BOF includes the protein amino acid composition, lipid profiles, pigment content, cell wall composition, carbohydrate content, DNA nucleotide fraction, RNA nucleotide fraction, and mineral and trace element composition, for a total of 121 compounds. However, detailed biochemical composition data are lacking for intracellular metabolite concentrations in MED4, and our BOF lacked information on free nucleotides, free amino acids, and the soluble pool (BioPool) concentrations, which were instead taken from the more completely characterized cyanobacterium *Synechocystis* sp. PCC6803. Cumulatively, these three pools make up less than 5% of ash-free dry weight (DW) and correspond to 4% of the variance of the growth rate under optimal growth, mostly (59%) due to spermidine and nicotinamide dinucleotides (see Data Set S2 in the supplemental material). Growth-associated maintenance (GAM) and non-growth-associated maintenance (NGAM) ATP requirements were calculated according to the method described by Feist et al. (56). The sensitivity of growth rate to alterations in the biomass composition (Ψ*_k_*) was evaluated by brute force, analogous to the calculation of shadow prices, as follows:

(6) $$\begin{array}{c} \psi_{k}=\frac{dZ}{dX_{k}^{\mathrm{BIO}}},\\ X_{k}^{\mathrm{BIO}}=S_{\mathrm{BIO}}+a_{k}^{T}S_{\mathrm{BIO}} \end{array}$$

where $X_{k}^{\mathrm{BIO}}$ is the biomass equation *S*_BIO_ with variable composition *k*. This is accomplished by varying either a pool of biomass precursors, where BIO is the index of the biomass reaction and *a_k_* is a vector of ones with an element of variable magnitude corresponding to a crude fraction (e.g., protein), or a specific compound within that crude fraction (e.g., l-lysine). These targeted elements for each biomass precursor pool or compound were varied by an arbitrarily small interval (−1 ppm ≤ Δ*a_k_* ≤ 1 ppm), and FBA was then performed to quantify the resulting change in growth rate (Δ*Z*).

An exhaustive search algorithm was implemented to quantify the change in growth rate as a function of varying biomass precursor pool compositions that satisfied a particular carbon/phosphorus molar ratio. In this way, equation 1 is additionally subject to the following:

(7) $$\begin{array}{c} e_{m}^{\mathrm{BIO}}\in \left\{Q_{\min,m}^{C/P}\leq Q_{m}^{C/P}\leq Q_{\max,m}^{C/P}\right\}\\ Q_{m}^{C/P}=\frac{\sum_{l=1}^{L}\sum_{k=1}^{K}a_{kl}Q^{C}}{\sum_{l=1}^{L}\sum_{k=1}^{K}a_{kl}Q^{P}} \end{array}$$

where *Q*^*C*^ is the number of carbon atoms and *Q*^*P*^ is the number of phosphorus atoms of each compound (*k*) in each biomass precursor pool (*l*) and $e_{m}^{\mathrm{BIO}}$ refers to the *m*^th^ target C/P composition ($Q_{m}^{C/P}$), derived from the elemental matrix *E*, within an interval 10% below $Q_{\mathrm{min,}m}^{C/P}$ and above $Q_{\mathrm{max,}m}^{C/P}$. Table 2 summarizes the BOF pool composition and sensitivity; details of composition and sensitivity under carbon-, light-, phosphorus-, and nitrogen-limited growth conditions are provided in Data Set S2 in the supplemental material.

**TABLE 2:** Crude biomass composition and growth sensitivity of *i*JC568^a^

Component	Composition (% of total DW)	Ψ (% of total)
DNA	1.2	<1
RNA	4.7	2
Protein	58.1	41
Lipid	11.5	35
Pigments	3.8	5
Cell wall	5.0	5
Carbohydrate	2.9	7
Free nucleic acids	<0.1	<1
Free amino acids	2.1	<1
BioPool	2.9	3
Minerals and trace metals	2.4	<1

a DW, ash-free dry weight; Ψ, growth sensitivity.

### Culture conditions and analytical procedures.

Axenic *Prochlorococcus marinus* strain MED4 (courtesy of S. W. Chisholm) was grown in 30-ml batches in 70-ml borosilicate glass tubes in modified PRO99 low-nutrient-seawater-based medium (31). P-limited growth was achieved after three transfers into 2 µM H_2_PO_4_^−^, with a resulting N/P ratio of 200. Cells were grown at 24°C under cool white fluorescent light programmed to a parabolic 14-h/10-h light/dark cycle reaching a peak irradiance of 45 µmol photons m^−2^ s^−1^. Cell growth and contamination were monitored daily by flow cytometry (57). Cells were harvested by centrifugation (14,000 × *g*), and the pellets resuspended in 100 µl of 0.2-µm-filtered seawater containing 0.2% paraformaldehyde. Aliquots of harvested cells were allowed to fix in the dark at 4°C for 30 min prior to analysis of lipopolysaccharide by the *Limulus* amebocyte lysate spectrophotometric method (58).
