# Supplementary figures and images for: Adaptive Evolution of Phosphorus Metabolism in Prochlorococcus
Source: mSystems. 2016 Nov 15;1(6):e00065-16. doi: 10.1128/mSystems.00065-16 (PMC5111396; doi:10.1128/mSystems.00065-16)

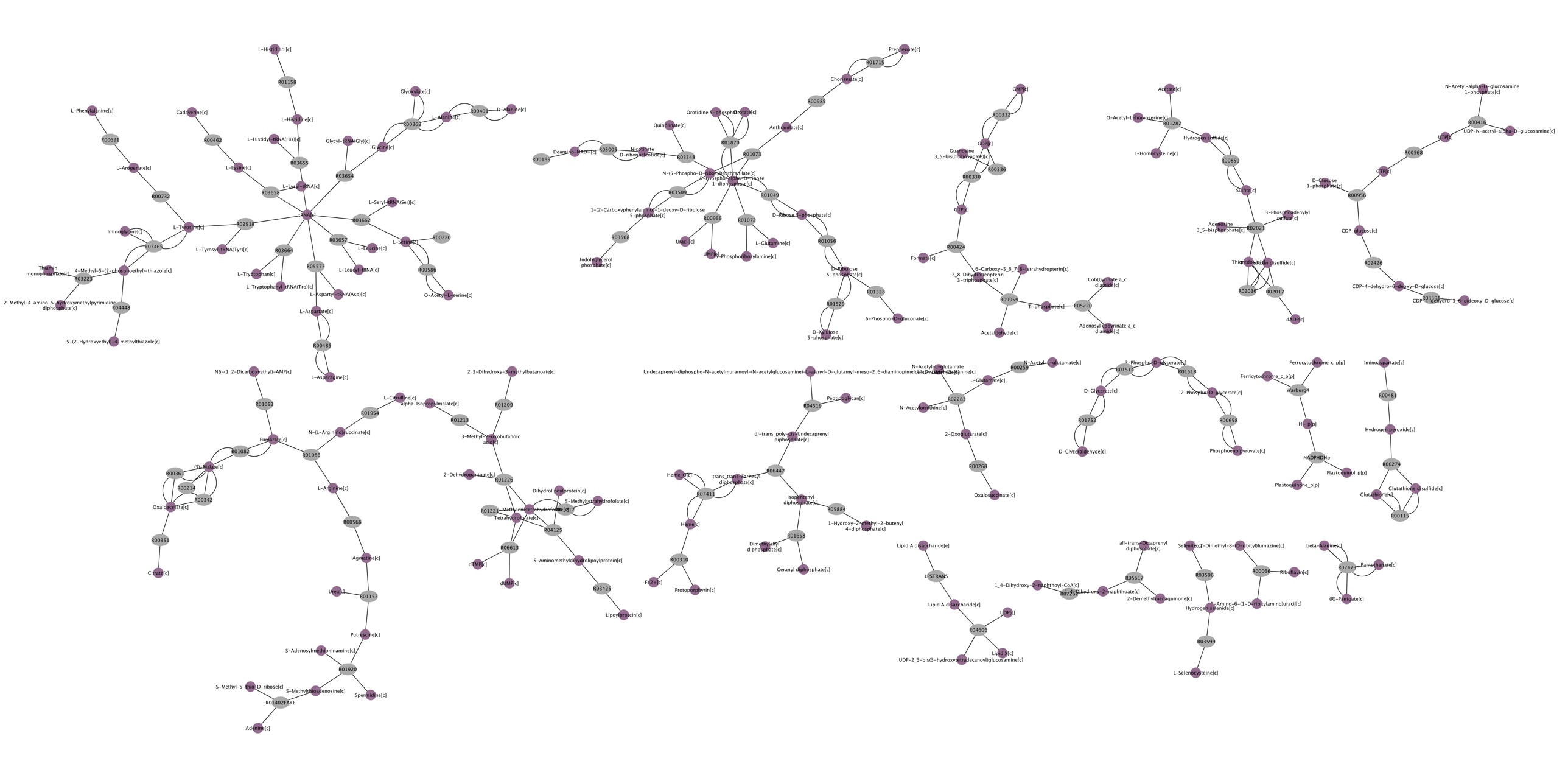

Supplement: Figure S3 [file sys006162062sf3.jpg]

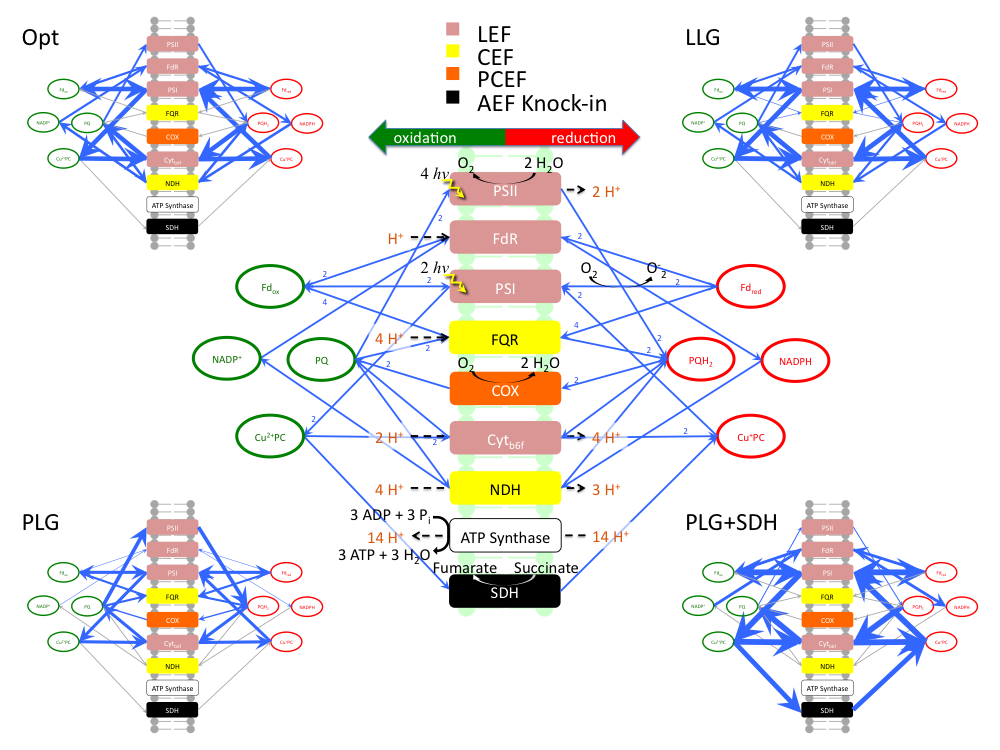

Supplement: Figure S4 [file sys006162062sf4.tif]
